# Oversulfated Chondroitin Sulfate Inhibits the Complement Classical Pathway by Potentiating C1 Inhibitor

**DOI:** 10.1371/journal.pone.0047296

**Published:** 2012-10-15

**Authors:** Zhao-Hua Zhou, Mohsen Rajabi, Trina Chen, Elena Karnaukhova, Steven Kozlowski

**Affiliations:** 1 Division of Monoclonal Antibodies, Office of Biotechnology Products, Office of Pharmaceutical Science, Center for Drug Evaluation and Research, Food and Drug Administration, Silver Spring, Maryland, United States of America; 2 Division of Hematology, Center for Biologics Evaluation and Research, Food and Drug Administration, Silver Spring, Maryland, United States of America; University of Leicester, United Kingdom

## Abstract

Oversulfated chondroitin sulfate (OSCS) has become the subject of multidisciplinary investigation as a non-traditional contaminant in the heparin therapeutic preparations that were linked to severe adverse events. In this study, it was found that OSCS inhibited complement fixation on bacteria and bacterial lysis mediated by the complement classical pathway. The inhibition of complement by OSCS is not due to interference with antibody/antigen interaction or due to consumption of C3 associated with FXII-dependent contact system activation. However, OSCS complement inhibition is dependent on C1 inhibitor (C1inh) since the depletion of C1inh from either normal or FXII-deficient complement plasma prevents OSCS inhibition of complement activity. Surface plasmon resonance measurements revealed that immobilized C1inhibitor bound greater than 5-fold more C1s in the presence of OSCS than in presence of heparin. Although heparin can also inhibit complement, OSCS and OSCS contaminated heparin are more potent inhibitors of complement. Furthermore, polysulfated glycosaminoglycan (PSGAG), an anti-inflammatory veterinary medicine with a similar structure to OSCS, also inhibited complement in the plasma of dogs and farm animals. This study provides a new insight that in addition to the FXII-dependent activation of contact system, oversulfated and polysulfated chondroitin-sulfate can inhibit complement activity by potentiating the classical complement pathway regulator C1inh. This effect on C1inh may play a role in inhibiting inflammation as well as impacting bacterial clearance.

## Introduction

Oversulfated chondroitin sulfate (OSCS), a member of the family of glycosaminoglycans (GAGs) which includes, heparin, heparan sulfate, dextran sulfate, chondroitin sulfate A (CS-A), CS-B, CS-C, CS-E and their oversulfated forms, was found to be a major contaminant in heparin during the period of time in 2007–2008 with increased heparin adverse events [1]. Clinical symptoms induced by OSCS-contaminated heparin included: hypotension, nausea and shortness of breath within 5 to 10 minutes after intravenous injection of the drug [1], [2]. *In vitro* studies indicated OSCS can activate the contact system with Factor XII (FXII)-dependant activation of the kinin-kallekrein system and generation of anaphylatoxins C3a and C5a [1]. Further studies confirmed that kallekrein induced by OSCS generated bradykinin, a mediator that can increase vascular permeability and thus explain the observed clinical symptoms [3]. Although anaphylactoid factors C3a and C5a increased, the generation of C3a and C5a bypassed any known complement activation pathways. As GAGs have interactions with a variety of plasma proteins including complement components [4], such as heparin potentiation of C1 inhibitor binding to C1-esterase, it is important to assess whether OSCS has any impact on complement activation pathways.

Complement can be activated by a number of mechanisms, including the classical complement pathway, the alternative complement pathway, and the mannose-binding lectin pathway, each comprised of several functional units [5], [6]. Activation of complement may have two distinct biological consequences: One is the irreversible structural and functional alterations of biological membranes leading to cell death (lysis), and the second is the activation of specialized cell functions (opsonization, chemotaxis).The classical pathway is activated by IgG- and IgM-type complexes and involves 11 proteins that have been grouped into three functional units, recognition, activation and membrane attack. The recognition unit consists of C1q, C1r and C1s. The activation unit consists of C2, C3, C4 and the membrane attack unit comprises of C5, C6, C7, C8, and C9. The alternative pathway bypasses C1, C2, and C4 and acts on C5-9 in a manner analogous to that of the classical pathway mechanism [7], [8]. The lectin pathway is homologous to the classical pathway, but initiates with the opsonin, mannose-binding lectin (MBL), and ficolins, instead of C1q [9], [10]. Because of the overlapping components, assessments of the classical pathway activation are generally used to test complement function [11].

The interactions of OSCS with the complement system may lead to either inhibition or enhancement of complement function in host responses to infections [12], [13] or in other diseases involving complement activation. This would include certain autoimmune diseases such as rheumatoid arthritis [14] . OSCS may have direct or indirect effects on complement. OSCS induced FXII-dependent generation of C3a and C5a in the plasma with the resultant anaphylactoid and chemotactic functions. The generation of C3a and C5a would also consume the complement components C3 and C5, and the depletion of these components may impact complement activation pathways. A more direct interaction of OSCS with complement components has been demonstrated using surface plasmon resonance [4] and this binding may impact complement activation. Another indirect effect of OSCS could be mediated through an interaction with regulators of the complement system. For example, previous studies have shown complement activation can be regulated by heparin and related GAGs through the complement regulator, C1inh. Heparin has been shown to potentiate the inhibition of C1s by C1 inhibitor by 15- to 35-fold, leading to decreased formation of C3 convertase in assays performed either with purified complement proteins or in whole serum [15]. However, a study using surface plasmon resonance did not reveal a difference in heparin and OSCS binding to complement components [4]. It is important to further investigate the comparative effects of heparin and OSCS on the complement pathways.

In the present study we investigated the interaction of OSCS with the complement classical pathway using a biologically relevant functional model as well as surface plasmon resonance. Although OSCS-contaminated heparin is unlikely to appear in the future due to current regulatory expectations, a related product, polysulfated glycosaminoglycan (PSGAG), is an approved veterinary medicine. The effect of PSGAG on complement activity in animal plasma was also investigated in this report.

## Results

### Oversulfated Chondroitin Sulfate Inhibits the Complement Classical Pathway

**Figure 1:**
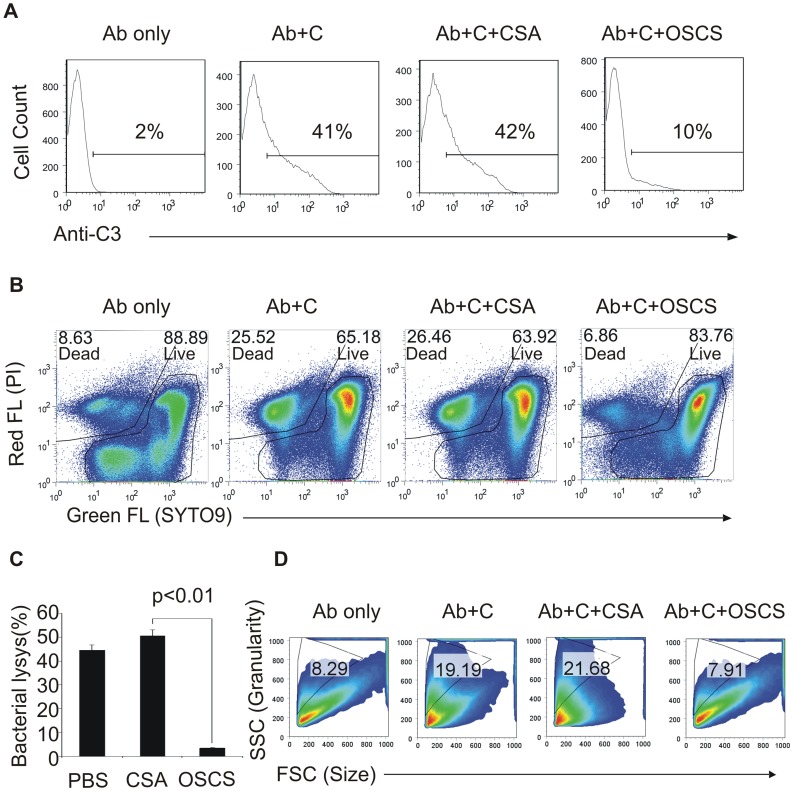
OSCS inhibits the complement classical pathway. *E. Coli BL21* bacteria were incubated with the polyreactive monoclonal antibody 2E4, followed by incubation with human complement plasma in the presence of PBS (positive control), 20 µg/ml of CSA or OSCS as described in Materials and Methods. (A) Complement fixation on bacteria was determined by PE-labeled anti-C3 monoclonal antibody and flow cytometry; (B) bacterial killing by complement was determined by flow cytometry using an SYTO9/PI staining bacterial viability kit and dead and live bacterial populations were gated with the percentages as indicated; (C) bacterial lysis was determined by ^3^H-dR release; (D) bacterial morphological changes were evaluated by FSC (proportional to cell size) and SSC (proportional to cell granularity). Data are representative of three independent experiments.

Natural polyreactive antibody clone 2E4 can recognize *E. Coli BL21* bacteria and induce bactericidal lysis through the complement classical pathway [16]. To test if OSCS impacts the complement classical pathway, OSCS or CSA were added to this model of the complement classical pathway activation system. Complement fixation on the bacteria, as determined by C3 deposition, was dramatically inhibited by OSCS but not by CSA (Figure 1A). Quantitative bacterial killing was determined by Live/Dead staining, and as shown in Figure 1B, 25.5% of bacteria were dead after addition of antibody and complement and 26.5% of bacteria were dead if the complement was treated with CSA. However, the percentage of dead bacteria was reduced to a baseline level of ∼8% if the complement was treated with OSCS. Bacterial lysis was also determined by ^3^H-TdR release in addition to Live/Dead staining. OSCS inhibited the bactericidal activity mediated by natural antibody clone 2E4 through the complement classical pathway (Figure 1C). Flow cytometry indicated that bacterial morphology changed after incubation with antibody 2E4 and complement. FSC (proportional to cell size) decreased while SSC (proportional to cell granularity) increased, indicating cell death. These morphological changes occurred in the CSA-treated sample as well as in the complement-treated positive control but were not observed in the OSCS-treated sample (Figure 1D).

### OSCS does not Block Antigen-antibody Binding in the Complement Classical Pathway

**Figure 2:**
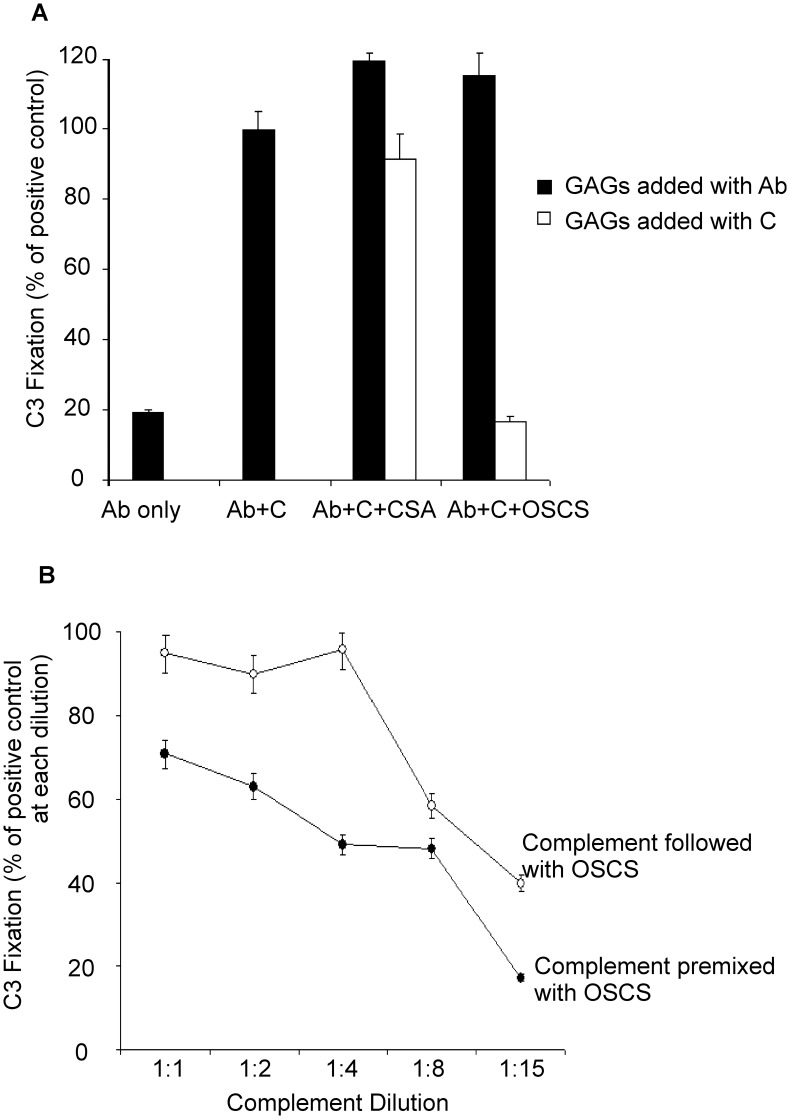
OSCS does not block the antigen/antibody interaction. (A) *E. Coli BL21* bacteria were incubated with CSA or OSCS -treated monoclonal antibody 2E4, then washed and incubated with complement plasma (▪); or antibody 2E4-treated *E. Coli BL21* bacteria were incubated with CSA or OSCS-treated complement plasma (□). C3 fixation was determined by Anti-C3 binding using flow cytometry and expressed as a percentage of C3 fixation in the absence of GAGs (PBS positive control); (B) Different dilutions of complement plasma were added to 2E4-treated *E. Coli BL21* bacteria followed by the addition of 20 µg/ml of OSCS (○), or 2E4-treated bacteria were added to different dilutions of complement plasma that had been pre-mixed with 20 µg/ml of OSCS at 37°C for 5 minutes (•). Complement C3 fixation was determined by anti-C3 monoclonal antibody binding using flow cytometry. The percentage of C3 fixation was calculated as percentage of mean fluorescence intensity of samples without OSCS at a comparable complement plasma dilution. The abbreviation Ab indicates monoclonal antibody 2E4, C indicates complement plasma. Data are representative of three independent experiments.

Antibody/antigen binding is the first step that initiates the complement classical pathway. To rule out the possibility that OSCS interferes with antibody binding to bacteria rather than blocking the activation of complement components, OSCS or CSA were added to bacterial samples together with the natural antibody 2E4. Before the addition of complement, the bacteria were washed with PBS to remove any unbound reagents. As shown in Figure 2A, the presence of OSCS during antibody/antigen interaction step did not block complement fixation while the presence of OSCS after the addition of the complement plasma dramatically inhibited C3 fixation (Figure 2A).

### Pre-treatment of Complement Plasma with OSCS Dramatically Enhanced Inhibition of Complement Fixation on Bacteria

Pre-incubation of the complement plasma with OSCS at 37°C for 5 minutes significantly decreased C3 fixation on bacteria in comparison to later addition of OSCS (Figure 2B). This indicates that OSCS interacted with some factor or factors in the plasma that inhibited the activity of complement. The decrease in the percentage of C3 fixation with a fixed dose (20 µg/ml) of OSCS was more notable at greater dilutions of complement (Figure 2B). The larger impact of a fixed dose of OSCS on diluted complement plasma may indicate that the ratio of complement components or other plasma factors to OSCS is critical for the inhibition effect.

### Consumption of C3 is not an Explanation of the Complement Inhibition by OSCS

**Figure 3:**
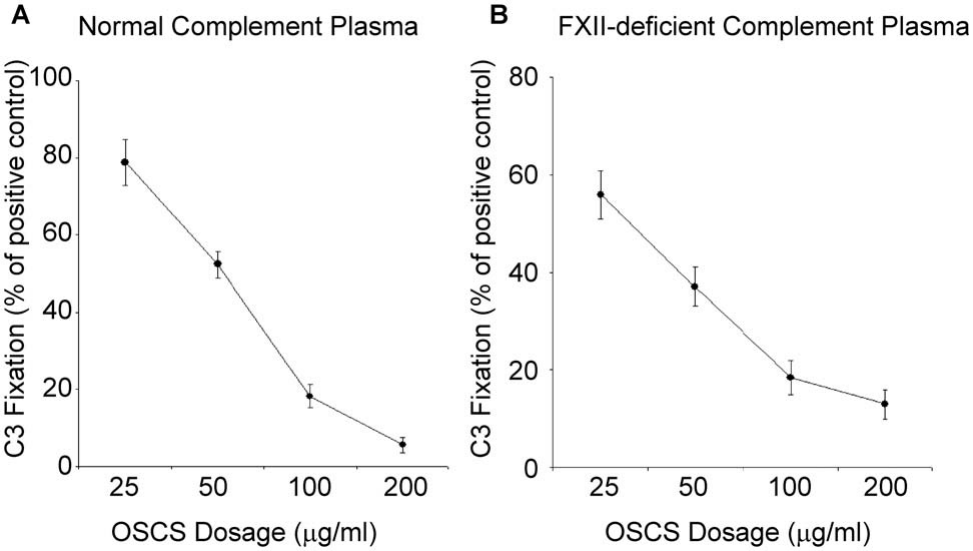
OSCS inhibits complement fixation in FXII-deficient plasma. 2E4-treated *E.Coli BL21* bacteria were incubated with different doses of OSCS in the presence of (A) normal human plasma or (B) FXII-deficient human plasma. C3 Fixation was determined by anti-C3 binding and flow cytometry, and the percentage of C3 fixation was calculated as percentage of mean fluorescence intensity of the PBS-treated positive control samples. Data are representative of three independent experiments.

The activation of FXII into FXIIa by OSCS generates kallikrein and further activates C3 and C5 into C3a and C5a [1], [4]. Thus, one explanation of the OSCS impact on C3 fixation and complement lysis would be the consumption of available C3 by OSCS. To test if this assumption is true, FXII-deficient plasma was incubated with OSCS. As expected, there was no C3a generation and therefore no C3 consumption (data not shown). However, the inhibition of C3 fixation by OSCS was still present as shown in Figure 3. This result indicates that FXII activation by OSCS and a resultant consumption of C3 is not the explanation for complement inhibition by OSCS.

### OSCS Inhibition of Complement Fixation on Bacteria is C1 Inhibitor-dependent

**Figure 4:**
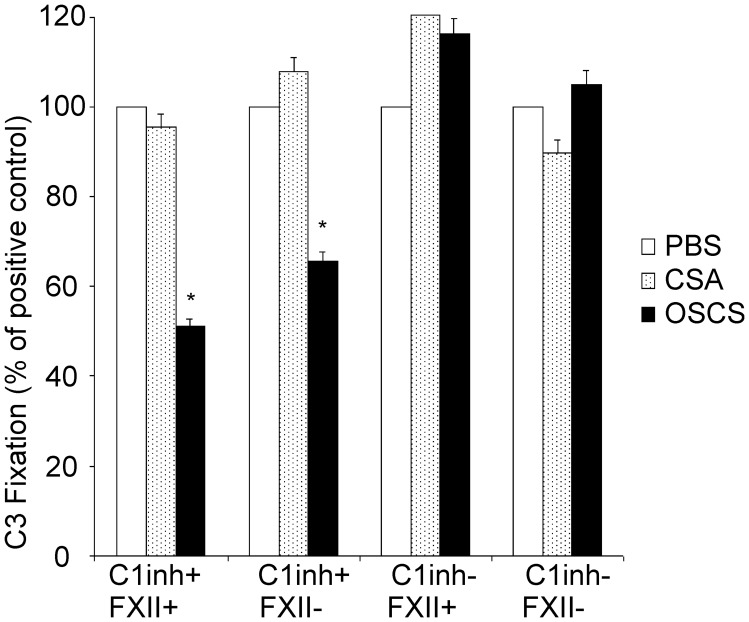
Complement inhibition by OSCS is C1 inhibitor dependent. 2E4-treated *E. Coli BL21* bacteria were incubated with normal (C1inh+/FXII+), FXII-deficient (C1inh+/FXII−), C1inh-deficient (C1inh−/FXII+), or C1inh and FXII double deficient (C1inh−/FXII−) complement plasma in the presence of PBS (positive control), 20 µg/ml of CSA (control GAG) or OSCS. C3 fixation was determined by anti-C3 binding using flow cytometry, and the percentage of C3 fixation calculated as percentage of mean fluorescence intensity of the PBS-treated positive control samples. A * indicates p<0.01 using the Student’s t test as compared with CSA-treated samples. This is significant at p<0.05 using the conservative Bonferroni correction for multiple comparisons. Data are representative of three independent experiments.

As described above, FXII activation and C3 consumption could not explain the OSCS inhibition of complement. In Figure 4, OSCS inhibits C3 fixation on bacteria with both normal (C1inh+/FXII+) and FXII-deficient (C1inh+/FXII−) complement plasma. However, when C1inh was depleted from either plasma, the OSCS inhibition disappeared (Figure 4). The control glycosaminoglycan CSA does not inhibit C3 fixation in any plasma tested. This result indicates that the inhibition of complement fixation by OSCS is dependent on C1inh.

### OSCS Enhances the Binding of C1 Inhibitor with C1s

**Figure 5:**
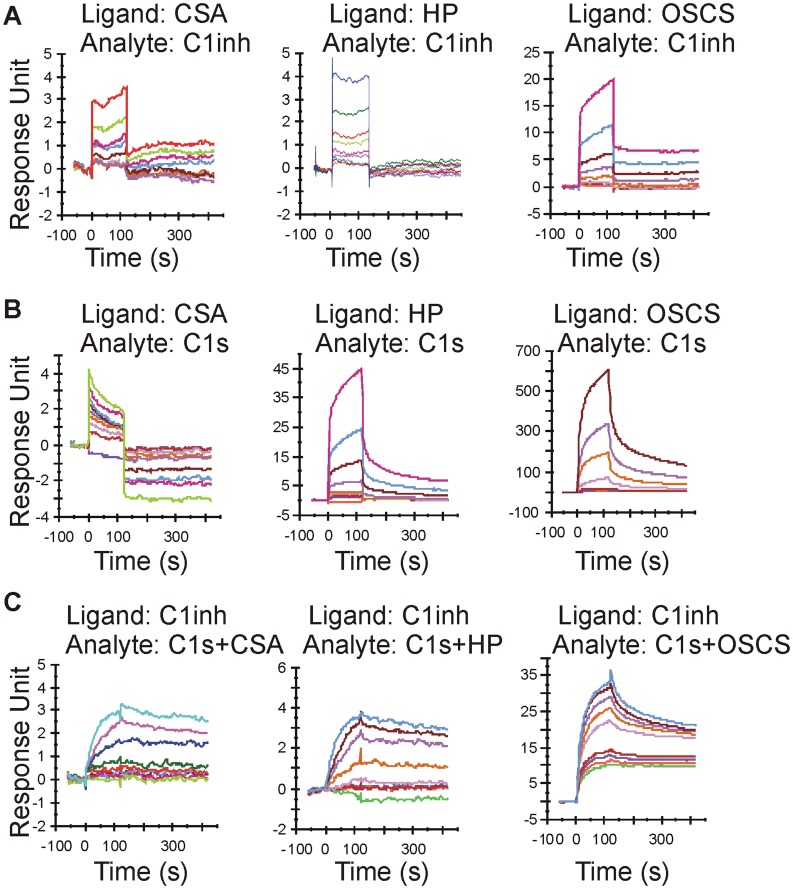
OSCS potentiates the binding of C1inh to C1s. The biotin-labeled GAGs, CSA, heparin and OSCS, were immobilized on NeutrAvidin-immobilized CM5 sensor chips as described in Materials and Methods. Biacore sensorgrams are shown for different analyte concentrations (from top 500, 250, 125, 62.5, 31.25, 15.6, 7.8, 3.9 and 1.95 nM) of (A) C1inh or (B) C1s binding to the immobilized GAG surfaces (ligands). The surfaces for the immobilized GAGs were regenerated each cycle using 2 M NaCl. (C) Biotin-labeled goat anti-human C1inh polyclonal antibody was immobilized on a streptavidin sensor chip and 1 µM of C1inh was injected to create a C1inh surface. Different concentrations of C1s (from top 1000, 500, 250, 125, 62.5, 31.25, 15.6, 7.8 and 3.9 nM) were then injected in the presence of 200 nM CSA, heparin or OSCS and the resulting sensorgrams are shown. The binding of GAGs (i.e., at 0 nM of C1s) with the C1inh surface were subtracted from the sensorgrams during data analysis. The C1inh surface was regenerated each cycle by injecting 30 µl/min pH 3.0 glycine for 30 seconds. Data are rep resentative of three independent experiments.

We have demonstrated C1inh is important in the OSCS mediated inhibition of complement. As the C1inh is the only inhibitor of C1s, we evaluated the interactions of both C1inh and C1s with OSCS and two other glycosaminoglycans, heparin and CSA. In order to evaluate these interactions using surface plasmon resonance, the GAGs were biotinylated and immobilized on NeutrAvidin-immobilized CM5 sensor chips to serve as surface ligands. As shown in Figure 5A, the direct interactions of C1inh with GAGs differ, with more C1inh binding OSCS than CSA or heparin (Fig. 5A). Figure 5B shows GAG interaction with C1s, with the highest binding (RU) for OSCS, followed by heparin and then CSA. The protein binding to GAGs may be due to a charge difference and may not impact the association of C1inh with C1s. To directly evaluate the impact of GAGs in the binding of C1inh with C1s, an SA sensor chip was immobilized with biotin-labeled anti-human C1inh antibody and then C1inh was captured generating a surface with C1inh as the ligand. Then different concentrations of C1s were dissolved in a low concentration (200 nM, ∼0.6 µg/ml) of CSA, heparin, or OSCS, and injected over the C1inh surface. As shown in Figure 5C, the binding of C1inh to C1s was dramatically increased in the presence of 200 nM of OSCS as compared to heparin or CSA.

### Heparin Lots Contaminated with OSCS have Increased Inhibition of the Complement Classical Pathway

**Figure 6:**
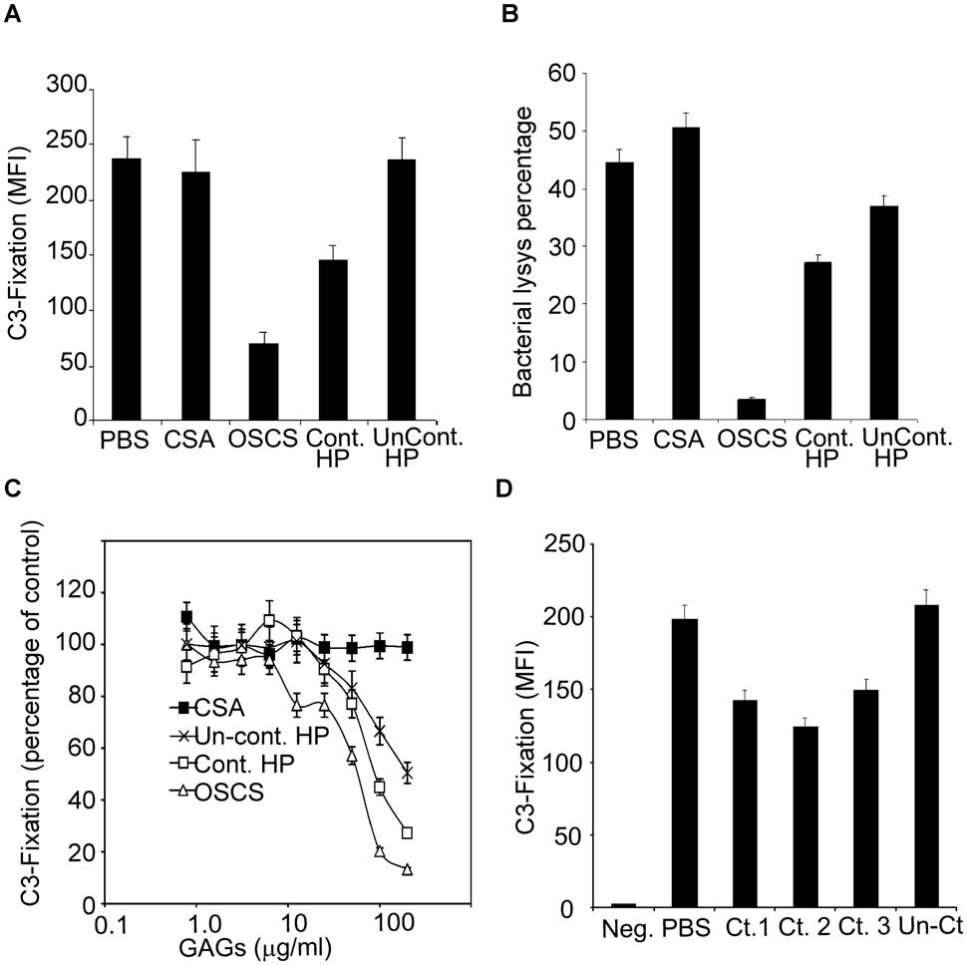
OSCS-contaminated heparin inhibits complement activity. (A) Polyreactive antibody 2E4-treated *E. coli* BL21 bacteria were incubated with complement plasma in the presence of 20 µg/ml of CSA, OSCS, contaminated heparin, uncontaminated heparin or a control buffer, PBS. Complement fixation on bacteria was determined by PE-labeled anti-C3 monoclonal antibody and flow cytometry; (B) The percentage of bacterial lysis was compared between complement plasmas in the presence of 20 µg/ml of CSA, OSCS, contaminated heparin, uncontaminated heparin or PBS. ^3^H-TdR release was used to determine the bactericidal activity of complement as described in Materials and Methods; (C) The percentages of C3 fixation on 2E4-treated *E. coli* BL21 bacteria, as compared to PBS-treated positive control samples, were evaluated after incubation with complement plasmas that had been pre-incubated with different doses of CSA, OSCS, contaminated heparin, and uncontaminated heparin. (D) Additional lots of heparin were evaluated for inhibition of complement activity as determined by C3 fixation. The abbreviation Ct. as well as Cont. indicates OSCS contamination. Data shown are representative of two to three independent experiments.

To check the impact of OSCS contaminated heparin on complement activation, natural polyreactive antibody-treated *E. coli BL21* bacteria were incubated with normal human plasma with the addition of PBS, CSA, OSCS, heparin from a lot contaminated with OSCS or heparin from an uncontaminated heparin lot. After incubation at 37°C for 5 minutes, bacteria were washed and complement component C3 fixation was determined by immunostaining and flow cytometry. As shown in figure 6A, OSCS had the largest inhibition on complement fixation, followed by OSCS-contaminated heparin, and there was no inhibition by uncontaminated heparin or CSA as compared with PBS, a negative control. Bactericidal activity was determined by ^3^H-TdR incorporation (figure 6B), and OSCS totally inhibited the complement classical pathway-mediated bacterial lysis. OSCS-contaminated heparin partially inhibited the lysis; uncontaminated heparin had less inhibition on the lysis, while there was no inhibition by CSA (figure 6B). Complement inhibition in response to different doses of GAGs is shown in figure 6C. There was a dose-dependent inhibition of complement fixation by GAGs with OSCS having the strongest inhibition followed by OSCS-contaminated heparin and then uncontaminated heparin. As shown in figure 6D, three heparin lots with OSCS contamination but not an uncontaminated heparin lot inhibited complement activity at the dose of 20 µg/ml.

Of note that at systemic levels of heparin for anticoagulation (from 1–5 µg/ml) [1], [17], it is unlikely that complement inhibition would be significant. However much higher systemic levels may occur shortly after bolus injections and local levels may remain higher for longer periods of time with routes of administration that may have a local depot such as subcutaneous, intra-articular or intramuscular administration. In such situations high levels of OSCS contamination may impact local complement activity at doses used for treatment.

### Polysulfated Glycosaminoglycan Inhibits Complement Activity in Farm Animal and Dog Plasma

Polysulfated glycosaminoglycan (PSGAG) is a widely prescribed veterinary medicine for the control of signs associated with noninfectious degenerative and/or traumatic arthritis of animal synovial joints. PSGAG is a semisynthetic glycosaminoglycan prepared by extracting glycosaminoglycans (GAGs) from bovine tracheal cartilage. The GAG present in PSGAG is principally chondroitin sulfate containing 3 to 4 sulfate esters per disaccharide unit, therefore structurally close to OSCS (4 sulfate esters per disaccharide unit).

**Figure 7:**
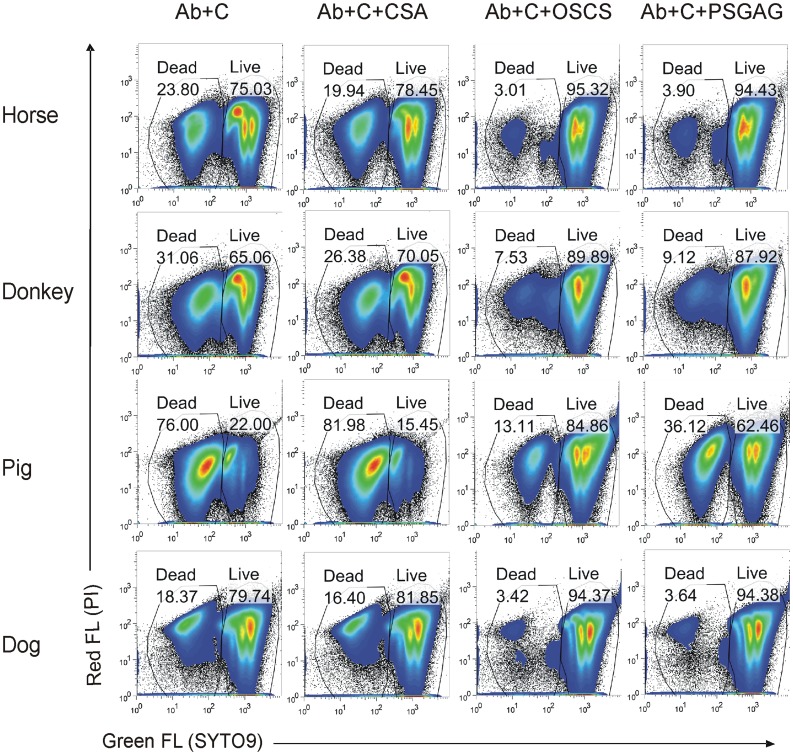
Polysulfated glycosaminoglycan (PSGAG) inhibits complement activity in animals. 2E4 antibody-treated *E. coli BL21* bacteria were incubated with complement from pooled horse, donkey, pig or dog plasma, or incubated with the same pooled plasma pre-treated with 20 µg/ml of CSA, OSCS or PSGAG. Bacterial viability was determined by an SYTO9/PI staining bacterial viability kit and flow cytometry. Dead and live bacterial populations and percentages are indicated. Data shown are representative of three independent experiments.

To evaluate the effect of PSGAG on complement activity, 20 µg/ml of PSGAG was used to treat complement-preserved plasma from horses, donkeys, pigs or dogs. PSGAG and control treated plasma samples were added to natural antibody 2E4-treated *E. coli* bacteria. Bacterial killing was tested by Live/Dead SYTO9/PI staining. As shown in Figure 7, 23.8% of bacteria were killed by natural antibody and horse complement and this number was reduced to 3.9% if the complement plasma was treated with PSGAG, indicating more than 80% inhibition. As expected, OSCS has similar complement inhibition and CSA has little if any inhibition. All the farm animal and dog plasmas tested have a similar pattern of inhibition (Fig. 7).

## Discussion

Glycosaminoglycans have been shown to interact with the complement system. Using a chromogenic assay for C1s activity and an ELISA to test the inhibition of complement C4 and C3 deposition on immobilized aggregated human Ig, Wuillemin et al reported [18] that GAG family members, including dextran sulfates with average MWs of 500,000 and 5,000 Daltons, heparin, heparan sulfate and CSA at concentrations from 100 to 1000 µg/ml could inhibit complement. Dextran sulfate with an average MW of 500,000 had the strongest inhibition. The inhibition was due to GAG enhancement of the second-order rate constant of the inactivation of C1s by C1inh [18], [19], [20].

OSCS was initially identified as a contaminant in certain lots of heparin that were associated with severe adverse events. Heparin is a polydisperse mixture of linear acidic polysaccharides, which is isolated by extraction from animal tissues, most commonly porcine intestines, and is a member of the glycosaminoglycan (GAG) family. OSCS had been previously prepared from chondroitin sulfate (CS), another member of the GAG family having similar backbone structure with heparin, by chemical sulfonation, and was shown to have anticoagulant activity [21]. Patients that received the OSCS contaminated heparin developed hypotension, shortness of breath and GI symptoms compatible with contact system activation [2]. In vitro studies showed OSCS activated contact system Factor XII (FXII) and induced kinin-kallikrein activation. OSCS also induced the generation of the anaphylactoid toxins C3a and C5a in a manner that bypassed the C3 and C5 convertases but was also dependent on FXII [1].

The impact of GAGs on complement and the observed effect of OSCS on complement components C3 and C5 suggested further evaluation of OSCS and complement. Investigation of the OSCS interactions with complement components (from C1 to C9) using surface plasmon resonance by Linhardt’s group [4] suggested that OSCS can bind to the complement components with moderate to high affinity comparable to that of heparin. Therefore a difference in the impact of OSCS-contaminated versus uncontaminated heparin is not explained by differences in binding to complement components.

The impact of OSCS on the functional complement activity was not performed yet, which became the aim of our study. In earlier work, the complement function has been tested in vitro using a variety of models such as the standard 50% hemolytic complement (CH50) assay, the enzyme immunoassay and the liposome immunoassay [22]. In this study, we used an established model of natural antibody mediated bacterial lysis through the complement classical pathway [16]. The murine monoclonal polyreactive antibody 2E4 can bind with bacteria *E. coli BL21*, fix complement, lyse bacteria and generate anaphylatoxin C5a. This is a relevant model to study the impact of OSCS, OSCS-contaminated heparin lots, un-contaminated heparin lots and other GAGs on the complement classical pathway.

Using this biologically relevant model, antibody mediated complement-dependant bacterial lysis, we demonstrated that OSCS can inhibit the complement classical pathway as indicated by lower level of C3 fixation on bacteria as well as decreased bacterial lysis. Initially, we hypothesized that C3 consumption might explain the decreased fixation of C3b on the antibody-treated bacteria. This hypothesis was consistent with the FXII-dependent OSCS induction of C3a as well as C5a. We ruled this out as the depletion of FXII from plasma did not decrease complement inhibition by OSCS. In addition, C3 is an abundant protein in normal plasma with reference values of 0.67–1.29 g/L [23], and thus unlikely to be consumed to levels that would impact C3 fixation.

In a separate study, we reported that complement regulator, C1 inhibitor is an important factor in susceptibility to OSCS-contaminated heparin associated adverse events [24] . In this study we found that the complement inhibition by OSCS was also dependent on C1inh, as inhibition by OSCS disappeared with the depletion of C1inh from plasma. Surface plasmon resonance assay showed OSCS increased the binding of C1 inhibitor with C1s protease, the initial component of the classical complement pathway. Therefore OSCS inhibits the complement classical pathway by potentiating the interaction of C1inh with C1s.

The GAG enhancement of the C1inh - C1s interaction as a direct cause of complement inhibition is consistent with the crystal structure of C1inh that was determined for the serpin domain of recombinant C1inh in its latent form [25]. Based on this structure, surface charge pattern, heparin affinity measurements, and docking of a heparin disaccharide, a heparin binding site is proposed in the contact area of the serpinproteinase encounter complex [25]. Beinrohr et al proposed that by binding to C1inh and neutralizing its positively charged surface patches the polyanions facilitate the C1inh-C1s interaction (a “sandwich” mechanism) [25]. This can explain how the inhibitory activity of C1 inhibitor toward proteases, such as C1s or activated factor XI, can be greatly enhanced by heparin and other glycosaminoglycans [18]. Our data support this model for the enhancement of C1inh - C1s interaction by GAGs that we observed in the current study, as well as in our earlier work [26].

Heparin lots contaminated with OSCS can inhibit complement activity *in vitro.* However the sustained levels of 10–20 microgram/mL are unlikely with intravenous dosing of heparin although subcutaneous administration [27] of contaminated heparin may have allowed for higher local levels of OSCS. A veterinary drug, polysulfated glycosaminoglycan (PSGAG) that is very similar in structure to OSCS is still used in animals and administered locally (e.g. intramuscularly). PSGAG is also a polysulfated chondroitin sulfate with 3 to 4 sulfate groups per disaccharide unit and is considered to be a disease-modifying veterinary drug for osteoarthritis. PSGAG is anti-inflammatory and many mechanisms have been postulated from preservation of joint glycosaminoglycans to inhibition of PGE2 synthesis, toxic oxygen radical generation, and complement activation. Studies have shown an impact of PSGAG at relatively higher doses on complement-mediated lysis of red blood cells [28], [29] without a clear mechanism of action.

Our *in vitro* experiments using bacteria as model indicate PSGAG is a very strong inhibitor of complement fixation of bacteria. The potentiation of C1inh interaction with C1s by OSCS can also explain the effect of PSGAG on complement lysis and provide a mechanism for studies suggesting an increased likelihood of infections with intra-articular injection of PSGAG and low levels of bacteria [30], [31].

Although there was an increase in the absolute numbers of infections reported during the 2007–2008 timeframe of the OSCS contamination, the relative numbers decreased [24] . It may be of value to further assess GAG related products including PSGAG, for infection related adverse events, although adverse event reporting has many limitations. Based on the concentrations heeded to inhibit complement, there may not be an *in vivo* effect unless high doses are administered locally (e.g. subcutaneous administration) rather than systemically.

There have been suggestions that glycosaminoglycans can be used to inhibit the complement activity in situations such as autoimmune diseases [32]. PSGAG treatment of animal arthritis is an example of such a use for a GAG. As PSGAG is not administered intravenously, a kallikrein mediated adverse effect, such as seen with OSCS contaminated heparin, may be less likely. A human version of such a product was marketed in Europe and withdrawn. Heparin has recently been shown to prevent fetal loss in a model of anti-phospholipid syndrome by inhibiting complement activation [33]. A more potent inhibition of complement, such as seen with OSCS, may be useful.

Although OSCS complement inhibition was demonstrated with the classical complement pathway, we also observed OSCS inhibition of Factor B after treatment with complement serum (data not shown). This indicates OSCS may also modulate the alternative pathway. The potential interactions between OSCS and alternative pathway factors (e.g., Factor B, Factor H and properdin) need further investigation.

Since OSCS activates the contact system in humans as well as inhibiting complement, it is unlikely to be used in the future for the purpose of complement inhibition. However, it is unclear whether the same structural attributes are responsible for both effects. Development of a GAG which separates the anti-complement activity from the pro-kallikrein activity of OSCS could be of value in treatment of inflammatory disease.

In conclusion, OSCS can inhibit the complement classical pathway by potentiating the binding of C1inh with C1s. This potentiation is much stronger with OSCS than heparin. A veterinary drug, PSGAG, has similar effects to OSCS on bacterial lysis by complement. C1inh potentiation may explain the anti-inflammatory properties of PSGAG as well as experimental studies showing an increased likelihood of infections with intra-articular injection of PSGAG and low levels of bacteria.

## Methods

### Materials

OSCS-contaminated and un-contaminated heparin lots were obtained by the FDA from Baxter Healthcare (1000 U/ml or 5000 U/ml in 10 ml and 30 ml vials) [34]. Synthetic OSCS and commercial veterinarian drug polysulfated glycosaminoglycan (PSGAG) were obtained from the Division of Pharmaceutical Analysis, FDA at St. Louis. Chondroitin sulfate A (CSA) was purchased from Sigma (St. Louis, MO). Purified human complement C1 esterase inhibitor (C1inh) was purchased from Sigma and EMD Chemicals USA (Gibbstown, NJ). Normal pooled human plasma was purchased from Innovative Research (Novi, Michigan). Purified human Factor XIIa and Factor XII deficient plasma supplied by Hyphen BioMed (France) was purchased through Innovative Research. Pooled complement-preserved plasma from horse, donkey, pig and beagle were purchased from Bioreclamation Inc (Westbury NY). Activated C1s was obtained from EMD chemicals USA (Gibbstown, NJ). Anti-complement component C1 inhibitor mouse monoclonal antibody was obtained from the AntibodyShop (Demark) and purified peroxidase-conjugated goat anti-human C1inh IgG was purchased from Cedarlane laboratories (Canada). Protein A/G-Sepharose was purchased from BioVision (Mountain View, CA). PE anti-human complement C3 monoclonal antibody was purchased from Cedarlane laboratories. Aamine-PEO3-biotin and NeutraAvidin were obtained from Pierce (Rockford, IL); Biotinylated GAGs were prepared as described by Li et al [4] .CM5 sensor chips, Streptavidin (SA) sensor chips, 1-ethyl-3-(3-dimethylaminopropyl)-carbodiimide hydrochloride (EDC), N-hydroxysuccinimide (NHS), ethanolamine–HCl, HBS–P buffer (0.01 M HEPES, pH 7.4, 0.15 M NaCl, 0.005% surfactant P20), acetate buffer (pH 5.0), Glycine (pH 2.5), NaOH (50 mM), NaCl (4 M) Mg_2_Cl_2_ (5 M) and deionized water were from GE Healthcare (Piscataway, NJ).

### Affinity Depletion of C1inh from Human Plasma

16 sterile micro tubes (Sarstedt Inc., Newton, NC) each with 0.5 ml Protein A/G Sepharose beads were washed with cold PBS twice and centrifuged at 10,000 g. To half of the tubes (designated as “Anti-C1inh beads”) 150 µg monoclonal mouse anti-human C1inh IgG and 1 mg of goat anti-human C1inh polyclonal IgGs were added while to the other half of the tubes (designated as “Control beads”) either same amount of PBS or irrelevant goat IgGs were added. The tubes were incubated with rotation at 4°C for 20 minutes followed by three washes with cold PBS. Then 1 ml of normal human plasma and of Factor XII-deficient human plasma were added to the “Anti-C1inh beads” or “Control beads” tubes. The tubes were mixed by rotation for 20 minutes at 4°C, followed by centrifugation at 10,000 g. The bead-treated plasma samples were then transferred to second tube with the same beads and the steps were again repeated for a total of four rounds resulting in control human plasma (C1inh+/FXII+), C1inh-depleted plasma (C1inh−/FXII+), Factor XII-deficient plasma (C1inh+/FXII−) and C1inh-/FXII- double deficient plasma. The plasma was then aliquoted and frozen at −80°C. C1inh levels in the plasma were determined by ELISA and more than 98% of C1inh antigen was removed by this method.

### Immunofluorescence Staining and Flow Cytometry Assays

To measure the binding of antibody, 2×10^7^ bacteria were preincubated with 1% BSA at 4°C for 30 min to block nonspecific binding sites. The bacteria then were incubated with mouse monoclonal polyreactive or monoreactive antibodies (50 µg/ml). Unless indicated otherwise, to measure the binding of complement, antibody-treated bacteria were incubated with a 1∶8 dilution of human plasma that had been preadsorbed by four rounds of incubation with the bacteria to be tested to eliminate any bacteria-binding immunoglobulin in the human plasma. This was followed by incubation with PE-labeled murine anti-human C3 monoclonal antibody and fixation with 4% paraformaldehyde. Fluorescence intensity was determined by FACSCalibur or LSRII (BD, San Jose, CA) and analyzed using FlowJo software (TreeStar Inc., Ashland, OR). To test the impact of GAGs on the complement classical pathway, different doses of GAGs or PBS control were incubated with complement plasma for 5 minutes at 37°C before adding to antibody-treated bacteria as described above.

### Bactericidal Assays

*E. coli BL21* bacteria were treated with the 2E4 antibody as described above and mixed with a 1∶8 dilution of complement from human or 1∶4 dilution of animal plasma that had been treated with GAGs and incubated at 37°C for 15 mins. The bacteria were then washed with cold PBS. Bacterial killing was determined by Live/Dead BacLight Bacterial Viability and Counting Kit (Molecular Probes, Inc. Eugene, OR) and analyzed by flow cytometry according to the manufacturer’s instructions. Bacterial killing was also determined by ^3^H-dR release as described before [16]. In brief, *E. coli* were radiolabeled by culturing in LB broth with 20 mCi/ml [6-^3^H] Thymidine (GE Healthcare, Piscataway, NJ). Radio-labeled bacteria (3×10^7^) were suspended in PBS-BSA, then incubated with polyreactive or non-polyreactive monoclonal antibodies (50 µg/ml) to which different dilutions of GAGs-treated human complement plasma were added. After incubation at 37°C for 10 minutes, the reaction mixtures were transferred to 96-well filtration plates (Millipore) and washed, and the cpm remaining on the filters was determined with a Microbeta 1450 Trilux liquid scintillation counter (Wallac, Gaithersburg, MD). Percent lysis was calculated as [(cpm of bacteria treated with PBS) - (cpm of bacteria treated with complement)/(cpm of bacteria treated with PBS)]×100.

### Preparation of the Neutravidin Sensor Chip

Neutravidin in sodium acetate 5 mM (pH 5) was immobilized on the surface of CM5 sensor chip following the BIAcore T200 Immobilization procedure for amine coupling. Approximately 15000 RU of neutravidin was immobilized on CM5 chip surface (Flow cells 2, 3 and 4). The surface was then blocked by injecting 1 M ethanolamine. 50 mM NaOH was then injected to wash off non-covalently bound neutravidin. Flow cell 1 was similarly treated with buffer in the absence of neutravidin (control).

### Immobilization of Biotinylated GAGs on Neutravidin Sensor Chip

The neutravidin sensor chip, described above, was pretreated with three 5 µL injections of 50 mM NaOH in 1 M NaCl, to remove any nonspecifically bound contaminants. 20-µL of biotinylated CSA, heparin or OSCS (500 µg/mL) in HBS-PE+ running buffer (flow rate, 10 µL/min) were injected in flow cells 2, 3 and 4, followed by a 10-µL injection of 50% isopropanol/50 mM NaOH/1 M NaCl. Flow cell 1 was similarly treated with buffer in the absence of biotinylated GAGs (control). Approximately 600 RU of biotinylated GAGs were immobilized in flow cells 2, 3 and 4.

### SPR Measurements of C1inh and C1s Binding to Immobilized CSA, Heparin and OSCS

SPR was performed on a BIAcore T200 (GE Healthcare, Uppsala, Sweden) using the above described sensor chip immobilized with GAGs. C1inh and C1s were diluted in PBS or HBS-EP+ buffer (GE Healthcare, Uppsala, Sweden). Different dilutions of C1inh or C1s in buffer were injected at a flow rate of 20 µL/min. At the end of the sample injection (120 s), the same running buffer was passed over the sensor surface to allow for dissociation over 180 s. After dissociation, the sensor surface was regenerated by injecting 2 M NaCl to remove all the bound proteins. The response was monitored as a function of time (sensorgram) and analyzed by Biacore T200 Evaluation software.

### SPR Measurements of C1inh Binding to C1s in the Presence of GAGs

SPR was performed on a BIAcore T200. The biotinylated goat anti-human C1inh polyclonal antibody (R&D Systems, Inc. Minneapolis, MN) was immobilized to flow cells 2, 3 and 4 in a streptavidin sensor chip. Flow cell 1 was treated with saturating amount of biotin and served as a control. The successful immobilization of anti-C1inh was confirmed by the observation of a 1000 resonance unit (RU) increase in the sensor chip. 40 µl of 1 µM C1inh diluted in HBS-EP+ buffer was injected to create the C1inh surface and the successful immobilization of C1inh was confirmed by observation of a 30 RU increase in sensor chip flow cells 2, 3 and 4. Different concentrations of C1s were diluted in HBS-EP+ buffer in the presence of 200 nM CSA, heparin or OSCS, and were injected at a flow rate of 30 µL/min. At the end of the sample injection (120 s), the same running buffer was passed over the sensor surface to facilitate dissociation for 180 s. After dissociation, the C1inh surface was regenerated each cycle by flowing 30 µl/min PH 3.0 glycine for 30 seconds over the surfaces. All the sensorgrams were processed using the double referencing method to eliminate the nonspecific binding from background contribution and buffer artifacts by subtracting signals from the reference flow cell (i.e., FC1) and from buffer blank injections (in this case, 200 nM GAG) [35]. FC2, 3 and 4 served as repeats. The response was monitored as a function of time (sensorgram) and analyzed using Biacore T200 Evaluation software.

### Statistical Analysis

Duplicate samples were evaluated and experiments were repeated at least three times. Data are presented as mean or mean ± standard error of the mean. All paired comparisons were subjected to two-tailed Student’s t tests. Significance was set at a p value of less than 0.05. All differences noted in experiments with multiple paired comparisons were significant at a p value of < 0.01.
